# Exploring Copy Number Variants in a Cohort of Children Affected by ADHD: Clinical Investigation and Translational Insights

**DOI:** 10.3390/genes16091020

**Published:** 2025-08-28

**Authors:** Federica Mirabella, Valentina Finocchiaro, Mariagrazia Figura, Ornella Galesi, Maurizio Elia, Serafino Buono, Rita Barone, Renata Rizzo

**Affiliations:** 1 Child and Adolescent Neurology and Psychiatric Section, Azienda Ospedaliera Universitaria Policlinico ‘G. Rodolico-San Marco’, Department of Clinical and Experimental Medicine, University of Catania, 95124 Catania, Italy; mirabella.federica.91@gmail.com (F.M.); valentina.5.11.finocchiaro@gmail.com (V.F.); rita.barone@unict.it (R.B.); 2 Oasi Research Institute-IRCCS, 94018 Troina, Italy; mfigura@oasi.en.it (M.F.); ogalesi@oasi.en.it (O.G.); melia@oasi.en.it (M.E.); fbuono@oasi.en.it (S.B.); 3 Department of Medicine and Surgery, “Kore” University of Enna, 94100 Enna, Italy

**Keywords:** attention deficit hyperactivity disorder, CNV, array-CGH, dysmorphism, cognitive functioning

## Abstract

**Background/Objectives:** Attention Deficit Hyperactivity Disorder (ADHD) is a common neurodevelopmental disorder frequently associated with other neuropsychiatric conditions, characterized by high clinical heterogeneity and a complex genetic background. Recent studies suggest that copy number variations (CNVs) may contribute to ADHD susceptibility, particularly when involving genes related to brain development, attention regulation, and impulse control. This study investigated the association between CNVs and ADHD phenotype by identifying patients with and without potential pathogenic CNVs. **Methods**: We evaluated 152 well-characterized ADHD pediatric patients through comprehensive clinical assessments, including dysmorphic features, brain MRI, EEG patterns, and cognitive testing. CNVs were identified using array Comparative Genomic Hybridization (array-CGH). Participants were classified as carrying potentially causative CNVs (PC-CNVs), non-causative CNVs (NC-CNVs), or without CNVs (W-CNVs) and statistically compared across clinical and neurodevelopmental measures. **Results**: CNVs were identified in 81 participants (53%), comprising 13 with PC-CNVs (8.5%) and 68 with NC-CNVs (44.7%). ADHD symptoms were pronounced across all groups, but PC-CNVs showed a higher burden of comorbidities, suggesting a stronger genetic contribution to ADHD complexity. Significant differences were observed in oppositional behavior, inattentive symptoms, brain MRI findings, and developmental language anomalies. Several CNVs involved genes previously implicated in neurodevelopmental disorders, supporting a potential genetic contribution to the clinical complexity of ADHD. **Conclusions**: This exploratory study supports the role of CNVs in ADHD susceptibility and highlights the value of genetic screening for understanding clinical variability. Larger studies are needed to clarify genotype–phenotype correlations in ADHD and to guide personalized clinical management.

## 1. Introduction

Attention deficit/hyperactivity disorder (ADHD) is a clinically heterogeneous neurodevelopmental disorder characterized by inattention, impulsivity, and hyperactivity [1,2,3]. Although it has for a long time been considered a childhood disorder, it is now established that impairing ADHD symptoms persist in adulthood in around 65% of cases [4,5], with broad impact on academic, occupational, psychosocial, educational, and emotional functioning and a cumulative risk of adverse life outcomes [6,7,8].

Epidemiological studies estimate a prevalence of 5.3% in school-aged children, with a progressive decline across the lifespan [1,3].

Population surveys reported that the male-to-female ratio is 2.4:1 [3,9,10]. Diagnosis typically occurs in late childhood but is often delayed in girls, especially those with inattentive presentation [3].

Following the guidelines of the Diagnostic and Statistical Manual of the American Psychiatric Association 5th edition (DSM-5) and the International Classification of Diseases 11th edition (ICD-11), diagnosis of ADHD is based on a clinical interview conducted with the parent and/or the patient that assesses the core symptoms of the disorder [3].

DSM-5 requires at least six impairing symptoms of inattention or hyperactivity-impulsivity in children, or five impairing symptoms for diagnosis in those aged 17 years or older. Symptoms must occur across multiple settings and must be present for at least 6 months and have an onset before the age of 12 years. Moreover, they must cause clinically meaningful impairment and not be better explained by another condition. ICD-11 omits numbered symptom lists and instead requires several meaningful symptoms from one of the three clusters (i.e., inattention, hyperactivity, and impulsivity).

Cognitive and functional deficits in ADHD have been linked to a smaller anterior cingulate gyrus and dorsolateral prefrontal cortex (DLFPC) as well as the reduction in activity of the frontostriatal region, which overall determine the clinical hallmarks of ADHD [5].

Neuroimaging data from the ENIGMA consortium revealed that children with ADHD exhibit reduced volumes in several brain areas, including the nucleus accumbens, amygdala, putamen, hippocampus, and overall intracranial volume, in comparison to children with obsessive–compulsive disorder (OCD) and autism spectrum disorder (ASD) [11,12,13].

Up to 70–80% of individuals with ADHD are estimated to have at least one comorbid mental condition across their lifespan [14], most commonly learning disorders, autism spectrum disorder, tic/Tourette syndrome, obsessive–compulsive disorder, developmental coordination disorder, depression and anxiety disorders, and oppositional defiant disorder [15]. The coexistence of these conditions, together with variability in symptoms, impairment, genetic background, and brain anomalies, contributes to the marked clinical heterogeneity of ADHD [3].

The complex etiopathogenesis of ADHD is primarily influenced by genetic factors, but environmental factors (e.g., prenatal and postnatal factors, such as maternal smoking and alcohol use and exposure to environmental toxins) also play a critical role [3]. Furthermore, very preterm and extremely preterm-born children have a higher ADHD risk than children born at term [16].

ADHD is one of the most heritable conditions among the psychiatric disorders, displaying a greater concordance in monozygotic twins instead of dizygotic and in siblings instead of the general population, meaning that genetic factors play a predominant role in the development of the disorder [5].

Early ADHD genetic studies focused on the research of variants in candidate genes that were supposed to play an etiopathogenetic role, such as genes involved in dopaminergic or noradrenergic transmission [17,18].

Genome-wide association studies reported that polygenic effects of many common variants, each exerting very small effects, strongly contribute to the etiopathogenesis of ADHD [17], with a heritability from common genetic variants such as single-nucleotide polymorphisms (SNPs) that are estimated at 14–20% frequency in the population [3,19].

The largest genome-wide analysis found 27 significant loci implicating 76 genes, many of which play a role in the early stages of brain development [3].

Candidate gene association studies reported numerous susceptibility genes correlated to ADHD aetiopathogenesis. Among these ones, the serotonin transporter gene (*5HTT*), the serotonin 1B receptor gene (*HTR1B*), the dopamine transporter gene (*DAT1*), the D4 and D5 dopamine receptor genes (*DRD4* and *DRD5*), brain-specific Angiogenesis Inhibitor 1-Associated Protein 2 (*BAIAP2*), solute carrier family 6 member 3 (*SLC6A3*), synaptosome-associated protein of 25 kD (*SNAP25*), and catechol-O-methyltransferase (*COMT*) have been identified [17,20].

Increasing studies on copy number variation (CNV) have shed light on the genetic background of ADHD. CNVs come from different mutational mechanisms, including DNA recombination, replication, and repair-associated processes, and may disrupt genes involved in key neurobiological pathways, including dopaminergic signaling, thereby contributing to clinical heterogeneity [21].

Rare copy number variations have been associated with increased risk for several psychiatric and developmental disorders, such as schizophrenia, ASD, Tourette syndrome (TS), developmental delay, and ADHD [22,23,24,25]. Some CNVs implicate genes with potentially relevant biological functions for ADHD (for example, *PRKN* is involved in dopamine regulation) [3]. Despite these advances, relatively few studies have combined systematic CNV analysis with detailed clinical characterization. This limits our understanding of how CNVs influence the phenotypic variability and comorbidity burden in ADHD.

According to this observation, the aim of the study was to explore the potential relationship between CNVs and clinical features in a deeply phenotyped pediatric ADHD cohort. Our exploratory study analyzed the frequency of pathological anomalies such as dysmorphic features, brain MRI and EEG alterations, comorbid conditions, and symptom severity by comparing patients with potential causative CNVs (PC-CNVs), non-causative CNVs (NC-CNVs), and without CNVs (W-CNVs).

## 2. Materials and Methods

### 2.1. Participants

This study was directed and performed at the Child and Adolescent Neurology and Psychiatry Unit of the Medical and Experimental Department of Catania University, with the collaboration of Oasi Research Institute-IRCCS, Troina, Italy.

A total of 152 patients, aged 4–17 (M:F 128:24), were enrolled for the study between 2020 and 2024. Inclusion criteria were established according to the following parameters: (i) occurrence of a clinical diagnosis of ADHD, according to the DSM-5 criteria; and (ii) execution of molecular analyses by array-CGH.

Exclusion criteria included (i) ADHD diagnosis without complete standardized assessment of cognitive functions and symptom severity.

Each participant was clinically evaluated for ADHD symptoms, cognitive functions, and associated comorbidities, such as autism spectrum disorder or intellectual disability.

### 2.2. Procedures

Medical history with a focus on positive family history for neurodevelopmental delay was acquired by the participants’ parents. All participants underwent neuropsychiatric evaluation for ADHD, related comorbidities, physical and neurological examination. Fasting blood and urine samples were collected for standard blood tests and to exclude the presence of inherited metabolic disorders. The presence of epileptic seizures and isolated electroencephalogram (EEG) abnormalities were assessed. Additionally, brain magnetic resonance imaging (MRI) using a 1.5 T scanner was examined to identify morphological signs of altered brain development, areas with abnormal white matter signal, and atrophic changes.

### 2.3. Clinical Assessment

All participants underwent neuropsychiatric evaluation for ADHD and related comorbidities.

ADHD symptoms were assessed using the Conners’ Parent Rating Scale (Revised): Short Form (CPRS–R:S) and the Child Behavior Checklist (CBCL). The CPRS-R gathers parental reports of childhood behavioral difficulties and includes summary scales that support ADHD and assess its severity [26]. T-scores for oppositional, inattentive, and hyperactive ADHD index subscales were considered. The CBCL is a broad-spectrum inventory that records behavioral and emotional problems and competencies in children aged 4–18, as reported by parents or parent surrogates [27,28]. T-scores of internalizing, externalizing, and total problems composite scales were used.

To assess cognitive and developmental levels, IQ was measured using the Wechsler Intelligence Scale for Children (WISC-IV) [29] and the Leiter International Performance Scale—Third Edition (Leiter-R) [30]. Intellectual disability (ID) was diagnosed when IQ was below 70.

ASD was evaluated using gold-standard standardized diagnostic tests, including the Autism Diagnostic Interview (Revised) (ADI-R) and Autism Diagnostic Observation Schedule (ADOS).

To assess learning abilities (reading, writing, and calculation), Italian standardized tests were administered, including the DDE-2 (Battery for the Evaluation of Dyslexia and Dysorthography 2) [31], AC-MT (for evaluating calculation and problem-solving abilities) [32], and MT (to assess text reading speed and accuracy) [33].

Finally, to assess comorbidity with Developmental Coordination Disorder (DCD), the Movement ABC-2 test [34] (Movement Assessment Battery for Children—Second Edition) was used.

### 2.4. Genetic Analysis

All patients were screened with array-comparative genomic hybridization assays (array-CGH).

DNA was isolated from peripheral blood lymphocytes drawn from the proband by standard procedures. Array-CGH analyses were performed according to the manufacturer’s protocol, using Agilent SurePrint G3 Human CGH Microarray kit 8 × 60 K (Agilent Technologies, Palo Alto, CA, USA), 41.5 Kb overall median probe spacing; Agilent SurePrint G3 Human CGH + SNP Microarray kit 4 × 180 K (Agilent Technologies, Palo Alto, CA, USA), 25.3 Kb overall median probe spacing; and Agilent SurePrint G3 Human CGH Microarray kit 4 × 180 K (Agilent Technologies, Palo Alto, CA, USA), 13 Kb overall median probe spacing.

The image of the array was acquired using the Agilent SureScan Dx Microarray Scanner G5761A (Agilent Technologies, Palo Alto, CA, USA) and analyzed with Agilent CytoGenomics software v.5.1.2.1 (Agilent Technologies, Palo Alto, CA, USA). Genomic positions of the rearrangements were assigned according to the public UCSC database hg19/GRCh37 of the human genome.

### 2.5. Array CGH Data Analysis and CNV Classification

“Potentially causative” deletions/duplications, “non-causative”, or potentially benign familiar variants were assigned based on the scientific literature and private and public databases, such as the Database of Genomic Variants (DGV, http://dgv.tcag.ca/dgv/app/home (accessed on 12 December 2024)), the Decipher database (http://decipher.sanger.ac.uk (accessed on 12 December 2024)), the database of human CNVs hosted by IRCCS Oasi Maria SS of Troina (http://gvarianti.homelinux.net/gvariantib37/index.php (accessed on 12 December 2024)), the database of human genomic structural variation (https://www.ncbi.nlm.nih.gov/dbvar (accessed on 12 December 2024)) and the OMIM catalogue (http://www.ncbi.nlm.nih.gov/omim(accessed on 12 December 2024)).

In this study, we classified as “potentially causative variants” (PC-CNVs) all CNVs listed in the OMIM database and associated with ADHD. Additionally, we included in the PC-CNV category those potentially causative CNVs with a less certain role in the disorder but which have been sporadically linked to ADHD in the literature or affect genes known to be associated with ADHD or other neuropsychiatric conditions.

Into the category of “non-causative” CNVs (NC-CNVs), we included variants of unknown significance (VOUS) that have either never been reported in the literature (unknown CNVs) or have not been associated with ADHD. This category also encompassed VOUS, likely benign or benign variants [35].

When a PC-CNV was identified, parental testing was requested to determine its origin and guide decisions regarding family management and future genetic counseling. In some cases, the origin of the imbalances could not be determined, or due to both parents’ refusal to undergo evaluation, in adoption scenarios, or in cases involving single-parent families where the available parent was not a carrier of the proband.

### 2.6. Statistical Approach

Patients with PC-CNV, NC-CNV, and W-CNV were statistically compared to evaluate the outcome of the presence or absence of CNVs on dysmorphic features, comorbidities, EEG anomalies, brain MRI characteristics, ID, the rate of epilepsy, CBCL, and CPRS-R T-scores.

Clinical features of participants were summarized using the average and the standard deviation (SD) for continuous data or count and percentage (%) for categorical data.

On this basis, statistical analyses on pairwise comparison were performed using one-way analysis of variance (ANOVA) or the Kruskal–Wallis test for continuous data, depending on data distribution, and chi-square metrics (χ^2^) or Fisher’s exact tests for categorical data, according to the assumptions of each test. To determine the data distribution, thus establishing the use of a parametric or non-parametric test, Kolmogorov–Smirnov analysis was applied.

A *p*-value < 0.05 was set to show statistical significance. When the χ^2^ test or ANOVA/Kruskal–Wallis test yielded significant results, post hoc pairwise comparisons were performed, and a Bonferroni adjustment was applied to correct for potential Type I error inflation. The new alpha level after Bonferroni correction was set to 0.017.

Data were analyzed using GraphPad Prism v.8 version 10.0.0 (GraphPad Software, San Diego, CA, USA).

## 3. Results

### 3.1. Genetic Findings

Out of 152 children with ADHD, 81 (65 males, 16 females) tested positive for CNVs (53.29%), and 71 patients (63 males, 8 females) emerged with no CNVs (46.71%).

Thirteen patients (8 males and 5 females) had PC-CNV (16.05% of all the patients with CNVs), and 68 (57 males and 11 females) had NC-CNV (83.95% of all the patients with CNVs).

In some participants, array-CGH analysis revealed more than one single CNV; therefore, a total number of 123 imbalances were detected (64 duplications and 59 deletions) (Table 1).

In total, 50 patients showed a single CNV, 22 showed two CNVs, 7 showed three CNVs, and 2 showed four CNVs.

Thirteen imbalances (nine deletions and four duplications) were categorized as pathogenic/causative CNVs (13.82% of all the CNVs): nine deletions were located on chromosomes 15q13.2-q13.3, 1p34.3, 16p12.2 (found in two patients), 9q33.1, 22q11.21 (found in two siblings), 1q21.1q21.2, and 6q26, and the four duplications were located on chromosomes Xp22.11p21.3, 22q11.21, 1q21.1, and 16p11.2 (Table 2).

Out of the 13 patients with PC-CNVs, four were adopted; therefore, the origin of the rearrangement was not possible to establish. In two patients, the rearrangements emerged de novo, while in three patients (two of those are siblings), the imbalances were paternally inherited. In two patients the rearrangements were maternally inherited, and in three patients, parents were not available for the analysis.

One hundred six CNVs (48 deletions and 58 duplications) were considered as non-causative CNVs (86.18% of all the CNVs).

### 3.2. Participants’ Characteristics

**Figure 1:**
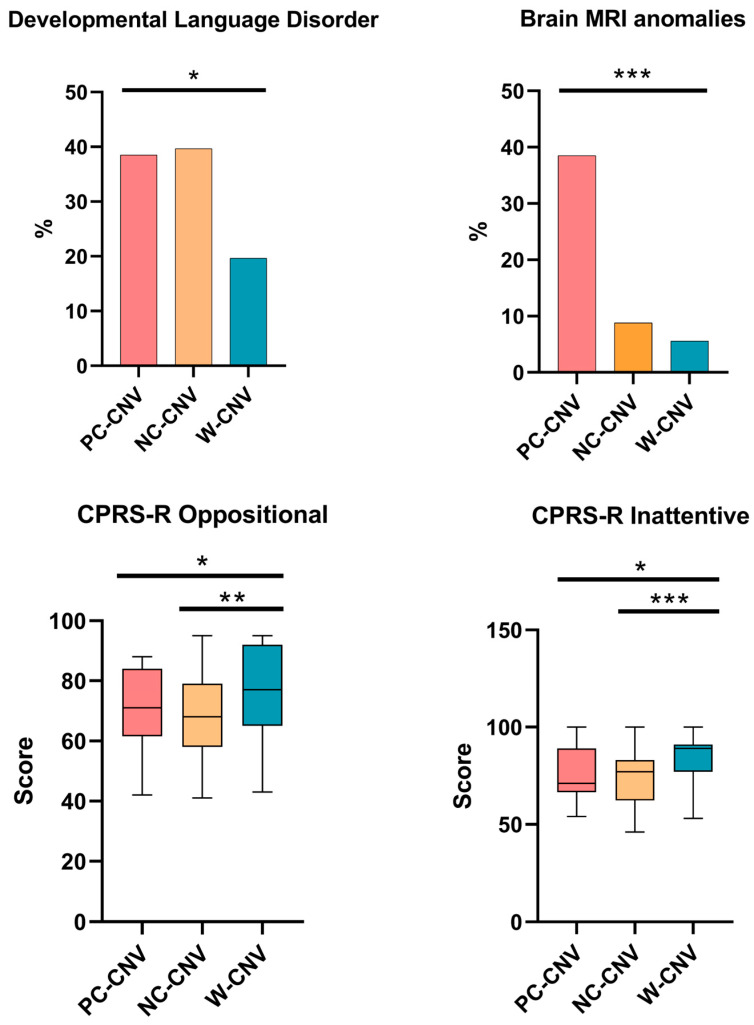
Clinical variables with statistically significant differences among ADHD children, according to CNV status. Paired post hoc comparisons with Bonferroni adjustment were used. * *p* < 0.05; ** *p* < 0.017; *** *p* < 0.008. CPRS-R Opp., Conners’ Parent Rating Scale (Revised), Oppositional; CPRS-R inatt., Conners’ Parent Rating Scale (Revised). PC-CNVs, causative copy number variants; NC-CNVs, non-causative copy number variants; W-CNVs, without copy number variants.

In this study, we recruited a total of 152 patients aged 4–17 years (mean age = 9 ± 2.5; male (M)/female (F) = 128:24; male = 84.1%), with a clinical diagnosis of ADHD, according to DSM-5 criteria. Table 3 shows the results of χ^2^ tests or ANOVA/Kruskal–Wallis analyses for the clinical and neuropsychiatric features of ADHD participants, categorized by CNV status. Significant differences in pairwise comparisons are displayed in Figure 1.

The most frequent neuropsychiatric comorbidity among all patients in our study was the developmental language disorder (DLD) (30.26%, *n* = 46), which was most of all observed in patients of the PC-CNV group (38.5%) and NC-CNVs group (39.7%), compared to patients without CNVs (19.7%) (Table 3), with a statistical significance (*p* = 0.0298).

The other most common comorbidities observed in our cohort of ADHD patients were tic disorder (26.97%) and specific learning disabilities (SLD) (26.31%), with a higher rate in PC-CNV patients (38.5% and 53.8%, respectively). The developmental coordination disorder (DCD) was observed in 39 participants (25.65%), while oppositional defiant disorder (ODD) was diagnosed in 35 patients (23% of all ADHD patients).

The mean full-scale IQ of all participants was 82.31 (±16.43 SD): 76.15 (±24.18 SD) for PC-CNVs patients, 82.32 (±16.47 SD) for NC-CNVs participants, and 83.46 (±15.68 SD) for W-CNVs patients.

Out of all the patients enrolled in our study, 31 (20.39%) showed intellectual disability (ID), with similar prevalence rates among the three groups. In 25 patients (16.44%), autism spectrum disorder (ASD) was comorbid with ADHD without significant relevance emerging between groups (*p* = 0.056). Borderline intellectual functioning (BIF) was detected in 17 patients (11.18%).

#### 3.2.1. Physical Measures

Seventy-two children with ADHD (47.37%) exhibited dysmorphisms, with a greater number of dysmorphisms observed during physical examination in the PC-CNV group (69.2%), compared to those observed in patients of the NC-CNVs (41.2%) and W-CNVs (49.3%) groups (Table 3). However, no significant differences have been revealed among the three groups (*p* = 0.162).

#### 3.2.2. Epilepsy and EEG Anomalies

Three patients (1.97%) had epilepsy that required medical treatment, one for each group. Isolated EEG anomalies (focal spikes, sharp waves, or slow waves) have been detected in 17 participants (11.18%). No significant differences were observed among patients with CNVs and patients without CNVs for the presence of epilepsy or isolated EEG anomalies (Table 3).

#### 3.2.3. MRI Studies

Brain MRIs were performed to detect brain structural abnormalities. Brain MRI anomalies were identified in 15 patients (9.86%), with significant discrepancies among the three groups (*p* = 0.001). Brain MRI abnormalities were more frequently observed in the PC-CNVs group (38.5%) compared to the NC-CNVs group (8.8%) and to the W-CNV group (5.6%) (Table 3, Figure 1).

#### 3.2.4. Behavioral Evaluation

Statistical analysis conducted on CBCL T-scores of the three composite scales (internalizing, externalizing, and total problems) has been identified in the clinical range for all the groups (CBCL Int. average T-score > 64; CBCL Ext. average T-score > 64; CBCL tot. average T-score > 67). However, no significant differences were observed compared to the three groups (Table 4).

Regarding the results of CPRS–R T-scores of the four subscales (oppositional, inattentive, hyperactive and ADHD index), there were statistically significant differences among groups concerning the oppositional and inattentive subscales (CPRS–R Opp. *p* = 0.011; CPRS–R Inat. *p* = 0.001), which were confirmed as statistically significant even after the Bonferroni correction (Table 4, Figure 1). Mean T-scores of the oppositional subscale were higher in the W-CNVs group (75.87 ± 15.05 SD), compared to the PC-CNVs (70.31 ± 13.78 SD) and NC-CNVs groups (68.5 ± 14.07 SD). The mean T-score of the inattentive subscale was higher in the W-CNVs group (83.21 ± 11.07 SD), compared to the PC-CNVs group (76.31 ± 15.09 SD) and to the NC-CNVs group (74.4 ± 14.41 SD). No significant difference emerged regarding mean T-scores of hyperactive and ADHD index subscales among the groups (Table 4).

## 4. Discussion

Extensive research has explored the association between risk CNVs and ADHD, given the complex genetic architecture of the disorder and the high range of variability. Several studies identified CNVs that may increase susceptibility to ADHD, mostly affecting genes involved in brain development and regions regulating attention and impulse control [36,37].

The aim of this exploratory study was to examine the impact of CNV occurrence in ADHD patients, analyzing their clinical significance and the potential relationship with typical somatic and neuropsychiatric features of ADHD.

In total, 81 of 152 patients enrolled in our study (53.29%) tested positive for CNVs. Thirteen children (16.04% of the total CNV patients) had potentially pathogenic CNV (PC-CNV). Clinical and molecular characteristics of the patients with PC-CNV are provided in Table 2 (probands 1–13).

Among these patients, one male subject (proband 1) with ADHD and intellectual disability exhibited a 1.9 Mb deletion on the long arm of chromosome 15, inherited from his father. This deletion spans several genes, including *MTMR10*, *OTUD7A*, and *CHRNA7*.

The child presented the following dysmorphic features: right posterior plagiocephaly, hypertrichosis on the back, high hairline, thick eyebrows, synophrys, long eyelashes, a flattened nasal tip, short philtrum, and low-set ears. Ziats et al. [38] described 18 patients with a 15q13.3 microdeletion, most of whom had intellectual disability, hyperactivity, attention problems, and externalizing behaviors associated with an ADHD phenotype, although no significant dysmorphic features were specifically linked to these patients. In contrast, deletions involving the *CHRNA7* gene, which encodes the alpha-7 nicotinic receptor highly expressed in the brain, are associated with phenotypic consequences such as intellectual disability, aggressive behavior, and ADHD, all typical of the 15q13.3 microdeletion syndrome phenotype [39].

Proband 2, a female with ADHD and moderate intellectual disability, exhibited a 240.9 Kb deletion on the short arm of chromosome 16, which includes the *OTOA* gene, associated with genetic hearing loss, and the *METTL9* gene, which is not linked to any specific disorders. While our patient does not have hearing loss, the deleted region overlaps with the critical region of the 16p12.2 microdeletion syndrome. Common features seen in individuals with this deletion include developmental delay, cognitive deficits, and psychiatric or behavioral challenges. In some cases, attention problems and aggression have also been reported [40].

Proband 3, a male with ADHD, moderate intellectual disability with speech impairment, oppositional defiant disorder, emotional disorder, and mild facial dysmorphisms, presented with a 470.7 Kb deletion in the 16p12.2 chromosomal region. This deletion affects several genes, including *UQCRC2*, *PDZD9*, *MOSMO*, *VWA3A*, *EEF2K*, *POLR3E*, and *CDR2*. The origin of this genetic imbalance could not be determined. This rearrangement is likely responsible for the 16p12.2 recurrent deletion syndrome, which, as previously mentioned for proband 2, is associated with variable clinical features such as developmental delay, cognitive impairment, psychiatric and behavioral issues, attention difficulties, and aggression [40].

Proband 4, a male with ADHD, speech disorder, and specific learning difficulties, exhibited a de novo 452.4 Kb microdeletion in the 1p34.3 chromosomal region and presented with mild dysmorphisms, including rounded facies, an anterior cowlick, and small eyes. The deletion in the 1p34.3 region encompasses several genes, including *KIAA0319L*, *NCDN*, *TFAP2E*, *PSMB2*, *CLSPN*, *AGO4*, and *AGO1*. Tokita et al. [41] described five children with large deletions at the 1p34.3 locus, including *AGO1*, *AGO3*, and sometimes *AGO4*, who showed developmental delay, language impairments, and facial dysmorphisms. Shalk et al. [42] reported 28 individuals with intellectual disability and de novo *AGO1* variants. Some of these probands exhibited autistic traits, aggressiveness, attention deficit/hyperactivity, and anxiety. *KIAA0319* is considered a candidate gene for reading disabilities [43]. This patient was previously reported as a case study in 2022 [44].

Proband 5 is a male affected by ADHD, Tourette syndrome, and learning disabilities. He exhibited several dysmorphic features, including a broad forehead, thick eyebrows, epicanthus, horizontal eyelid folds, low-set large ears, hypoplasia of the tragus, a flat philtrum, protruding columella, an ogival palate, a thin upper lip, V-shaped finger clinodactyly, and finger pads. The child carries a 2.8 Mb duplication on the 22q11.21 region (involving 86 genes, such as *PRODH*, *SCARF2*, and *HIRA*), though the inheritance pattern could not be determined due to his adoption. The phenotypic presentation of patients with proximal 22q11.2 microduplications is highly variable, with manifestations ranging from mild learning disabilities and subtle dysmorphic facial features to severe intellectual disability and multiple congenital malformations [45].

Wentzel et al. [46] reviewed 36 published cases of 22q11.2 duplication syndrome, noting that the most common features included intellectual disability/learning difficulties, memory deficits, impairments in perceptual organization and verbal comprehension, ADHD, and speech impairment (97%). Other observed characteristics included delayed psychomotor development (67%) and dysmorphic features, such as hypertelorism (70%), broad flat nose (53%), micrognathia (52%), velopharyngeal insufficiency (48%), dysplastic ears (45%), epicanthal folds (42%), and down-slanting palpebral fissures (41%).

Later, Lindgren et al. [47] described two patients with microduplications in the distal portion of chromosome band 22q11.2 with a diagnosis of ADHD.

Proband 6, a female with ADHD, borderline intellectual functioning, language and speech impairments, and EEG abnormalities, exhibited the following dysmorphisms: triangular facies, high forehead, small chin, and hyperlaxity of the small joints. The deletion on 9q33.1 encompasses the *ASTN2* and *TRIM32* genes. Astrotactin2 (*ASTN2*) plays a key role in regulating neuronal migration and synaptic strength through the trafficking and degradation of surface proteins. Deletions involving *ASTN2* have been identified in patients with schizophrenia, bipolar disorder, and autism spectrum disorder in copy number variant (CNV) analyses. Disruption of *ASTN2* is considered a risk factor for these neurodevelopmental disorders, including schizophrenia, bipolar disorder, autism spectrum disorder, and ADHD [48].

Proband 7, a male with borderline intellectual functioning, language disorder, and EEG abnormalities, exhibited a duplication in the 16p11.2 chromosomal region, which encompasses 30 genes, such as *PRRT2*, *PAGR1*, and *TAOK2*. As he is adopted, the origin of the genetic imbalance could not be determined. The phenotype associated with the 16p11.2 duplication is highly variable, ranging from asymptomatic cases to individuals with intellectual disability, motor delays, ADHD, and autism spectrum disorder (ASD) [49].

Proband 8, a female with ADHD, learning disabilities, and microcephaly, exhibited two CNVs: a 1.2 Mb deletion spanning 1q21.1q21.2 (with a known pathogenic role) and a 16p13.12 deletion of uncertain significance. As she is adopted, the origin of the genetic imbalance could not be determined. She presented with several dysmorphic features, including microcephaly, a small forehead, arched eyebrows, mild synophrys, horizontal palpebral fissures, a thin upper lip, and microretrognathia.

The deletion on chromosome 1 overlaps with the critical region of 1q21.1 microdeletion syndrome, which is associated with a variety of phenotypic features, including microcephaly, intellectual disability, developmental delay, and craniofacial dysmorphism [50,51]. Additionally, behavioral and psychiatric conditions such as ADHD, autism spectrum disorders (ASD), schizophrenia, and seizures have been reported in affected individuals [52].

Probands 9 and 10, a male and a female sibling pair, shared the same genetic imbalance, a single 22q11.21 deletion inherited from their father. Both siblings have comorbid Specific Learning Disabilities, with the male proband also diagnosed with a tic disorder. They display mild dysmorphic features, such as abnormal ears and a prominent forehead. Common characteristics of central 22q11.21 deletions include developmental delay, intellectual disability, language delay, dysmorphic features, and psychiatric or behavioral problems. The most frequent dysmorphic features observed in these patients were abnormal ears, upslanted palpebral fissures, and a prominent forehead [53], which were also seen in our two patients.

Proband 11, a male with ADHD, Tourette syndrome, and learning disabilities, exhibited an 805 Kb deletion on chromosome 6q26, inherited from his mother. A similar CNV was previously reported in a patient with Tourette syndrome and obsessive–compulsive disorder (OCD) in a large, genome-wide study of rare CNVs in OCD and TS [54]. This deletion includes the *PARK2* gene (OMIM: 602544), a neurodevelopmental gene initially associated with early-onset Parkinson’s disease. [55]. Jarick et al. [56] suggested that copy number variants at the *PARK2* locus may contribute to the genetic susceptibility of ADHD.

Proband 12, a female with ADHD, Tourette syndrome, and OCD, exhibited a deletion on chromosome 1 that overlaps with the critical region of 1q21.1 microdeletion syndrome. This syndrome has been associated with a range of phenotypic features, including microcephaly, intellectual disability, developmental delay, and craniofacial dysmorphism [50]. Additionally, behavioral and psychiatric conditions, such as ADHD, ASD, schizophrenia, and seizures, have been detected in a subset of patients with this condition [57,58,59].

Proband 13, a male with ADHD, tic disorder, learning disabilities, and microcephaly, exhibited no intellectual disability (IQ = 89) and a normal brain MRI. He carried three genetic imbalances, one of which is an Xp22.11p21.3 duplication with a pathogenic role, affecting the *ARX* gene. As he is adopted, the origin of the imbalances could not be determined.

The *ARX* gene (MIM 300382) is crucial for numerous functions, including neuronal stem-cell proliferation, migration, and differentiation; axonal guidance; and synaptic activity [60]. Clinical features associated with Xp21.3 duplication include syndromic intellectual disability (ranging from mild to severe), global developmental delay, hypotonia, autism, and hyperactivity [61]. However, Popovici et al. [62] reported two patients with Xp22.13 duplications, including *ARX*, who had normal intelligence.

The other two imbalances of uncertain significance include a 6q26 duplication, involving the *PARK2* gene, and an 11p15.4 duplication, encompassing the *TRIM21* and *TRIM68* genes.

While detailed clinical and molecular descriptions of these PC-CNV cases provide insights into genotype–phenotype correlations, the small number of patients limits broad generalizations. Each case illustrates how specific CNVs can contribute to diverse neurodevelopmental and behavioral outcomes, including intellectual disability, language impairments, and dysmorphic features, yet the heterogeneity of clinical presentations means that conclusions about causality or prevalence must be drawn with caution. Moreover, the variability in CNV size, gene content, and inheritance patterns adds further complexity, emphasizing that these findings are primarily descriptive and exploratory, and aim to highlight the potential mechanisms through which CNVs may influence ADHD phenotypes.

In line with this, several studies have reported an increased prevalence of large, rare CNVs in neurodevelopmental disorders, such as ASD [24], TS [23], and ADHD [22,36], highlighting their pathogenetic significance. Chromosomal microarrays (CMA) detected causative CNVs in an average of 12.2% of patients across 33 studies including 21,698 participants. Furthermore, ADHD patients show a 1.33 times higher rate of CNVs larger than 100 Kb with respect to healthy controls [63].

Trio-based studies more recently revealed that the overall variation rate of de novo CNVs was 4.6%, with respect to the 1.7% of previous studies.

The complex genetic architecture makes it difficult to identify ADHD-specific CNVs due to their increasing frequency and the overlap with CNVs also found in other neuropsychiatric disorders. Among the most reported CNVs associated with ADHD, several studies identified 15q13.1 duplications and 16p12.1 deletions [63], 16p13.11 duplications and 22q11.21 rearrangements [22], the latest of which was also detected in our analyses.

In this study, we conducted statistical tests on somatic and neuropsychiatric features to establish a potential relationship between CNV status and clinical severity, focusing on differences among groups of CNV patients.

Dysmorphic features were observed during physical examination, with a higher prevalence in PC-CNV patients (69.2%), compared to NC-CNVs (41.2%) and W-CNVs (49.3%). While physical anomalies can occur in individuals with ADHD, particularly when associated with certain genetic conditions, these traits are not specific to the disorder and may be present across a range of neurodevelopmental disabilities [64].

Comorbid conditions were most frequent in PC-CNV patients (100%) compared to NC-CNVs (97.05%) and W-CNVs (92.96%), with developmental language disorder (30.26%) being the most common.

This result aligns with findings in the literature, which indicate that speech impairments co-occur in approximately 40–50% of children with ADHD, and 20–30% of children with language impairments also exhibit ADHD [65]. Developmental language disorder (DLD) was observed at similar rates in both the PC-CNV and NC-CNV groups, and less frequently in patients without CNVs. ADHD and DLD share genetic risk loci, including *FOXP1* and *FOXP2*, with rare variants associated with both speech disorders and ADHD [66,67]. Both conditions are genetically heterogeneous, with CNVs and other variants contributing to a broader phenotypic spectrum [68].

Other frequent comorbidities included specific learning disabilities (26.31%), tic disorder (27%), oppositional defiant disorder (23%), developmental coordination disorder (25.65%), intellectual disability (20.39%), and autism spectrum disorder (16.44%).

Approximately 45% of children with ADHD meet the criteria for specific learning disabilities [69]. Tic disorder shows high comorbidity with ADHD, affecting over 50% of children with tics and around 20% of children with ADHD [70,71]. ADHD co-occurs with ASD in 20–50% of cases [72] and with Developmental Coordination Disorder in approximately 50% [73].

The clinical evaluation of comorbidity with epilepsy and isolated EEG showed a divergent low rate of patients (1.97% and 11.18%, respectively), though ADHD patients may have higher epilepsy prevalence than neurotypical peers [74]. Rare triple comorbidity involving ADHD, epilepsy, and Tourette syndrome has been reported [75], but children with epilepsy are often underdiagnosed or undertreated for ADHD [76].

In this study, brain MRI anomalies were identified in fifteen patients (9.86%), with a statistically significant difference among the groups. We observed a higher rate of Brain MRI anomalies in PC-CNV patients (38.5%), compared to NC-CNVs (8.8%) and W-CNVs (5.6%). Structural brain abnormalities have been identified in the brain MRIs of patients with ADHD compared to healthy controls. The ENIGMA study revealed that ADHD patients had significantly smaller volumes for the accumbens, amygdala, caudate, hippocampus, putamen, and ICV [12].

Regarding the neurobehavioral and emotional features, all groups showed T-scores within the typical ADHD clinical range for both the CPRS-R:S (T > 70) and CBCL (T > 64). In particular, results demonstrated a statistical significance between CNV groups for the CPRS-R:S oppositionality and inattention subscales, with higher scores in W-CNVs (75.87 ± 15.05 and 83.21 ± 11.07, respectively). This paradoxical finding highlights that the presence of CNVs is not the only factor influencing behavioral severity in ADHD. The elevated scores in W-CNVs may reflect the influence of multiple small-effect genetic variants, undetectable by array-CGH, as well as environmental and epigenetic factors contributing to symptom severity. These observations underscore the multifactorial and polygenic nature of ADHD, suggesting that both detected CNVs and other genetic or non-genetic factors collectively shape the cognitive and behavioral phenotype. Overall, the results of this study shed light on a range of rare deletions and duplications that involve key genes related to neurodevelopment of ADHD. We based it on a numerous cohort of study (N = 152), finding a good incidence of significant PC-CNV (16.05% compared to all positive CNVs).

Among ADHD risk rearrangements identified in this study, it is useful to report variants of uncertain significance that involve genes deserving to be mentioned and recurring in more than one patient.

In two patients, *BTBD9* gene variants (located in the chromosomal region 6p21.2) were found. Both patients exhibited a language disorder, with one of them also having a mild intellectual disability. *BTBD9* has been identified as one of the most common genetic risk factors for restless leg syndrome (RLS) [77]. The involvement of *BTBD9* in ADHD has also been reported in genome-wide association studies [78]. The presence of *BTBD9* variants in two patients from our cohort further supports the potential role of this gene in ADHD.

CNVs involving the *PARK2* gene, located in the 6q26 chromosomal region, were identified in four patients in our cohort, which overall had normal intellectual functioning. Two of these patients, in addition to ADHD, also had comorbid tic disorder and specific learning disabilities. Jarick et al. [56] suggested that CNVs in the *PARK2* locus may contribute to genetic susceptibility to ADHD. The presence of these gene variants in four of our patients (4.93% of all CNVs) further supports the involvement of *PARK2* in the genetic predisposition to ADHD.

In two patients, CNVs in *RBFOX1* (located in the chromosomal region 16p13.3) were found. These two individuals, in addition to ADHD, had an emotional and conduct disorder. This gene has been associated with autism and other neurodevelopmental disorders. Alterations in the *RBFOX1* gene have been found in various psychiatric and neurodevelopmental disorders that often are comorbid with anger, conduct disorders, and aggressive behaviors [79].

Overall, our exploratory study provides descriptive insight into the genetic basis of ADHD, underlining the complex genetic architecture, including cognitive and behavioral features, as well as any associated physical characteristics, and exploring the marked role of potentially pathogenic CNVs in the etiopathogenesis of the disorders.

## 5. Conclusions

As previously demonstrated in several studies, array-CGH genetic analysis is a valuable tool for identifying clinically relevant CNVs in patients with neurodevelopmental disorders (NDDs), including ADHD. In line with this evidence, our study allowed the identification of CNVs in 53.29% of patients with ADHD, and potentially pathogenic CNVs in 16.05% of total CNV-positive patients.

Many CNVs identified in this study involve genes that have already been associated with neurodevelopmental disorders, including ADHD, providing further evidence of their key role in the etiopathogenetic mechanisms. However, genetic factors alone are not often sufficient to explain the extreme phenotypic complexity of these disorders. A genetic diagnosis is not possible in most cases and should be considered primarily in selected cases where clinical features suggest a stronger likelihood of underlying genetic contributions. This includes patients with severe or early-onset symptoms, multiple neurodevelopmental comorbidities, atypical physical or developmental features, or a notable family history of neurodevelopmental disorders.

To further explore this, we investigated the relationship between CNV status (i.e., PC-CNV, NC-CNV, and W-CNV) and the common ADHD clinical features in a cohort of phenotypically well-characterized ADHD patients.

We found the typical ADHD clinical score range for all the groups, with statistically significant differences regarding brain MRI anomalies, CPRS-R oppositional, CPRS-R inattentive, and developmental language disorder. The latter is included in a broad spectrum of comorbidities that we have found associated with ADHD in our cohort. In particular, our study revealed higher rates of comorbid conditions in the PC-CNV group compared with the others, suggesting a greater influence that could make more intricate the clinical feature of ADHD in association with the genetic background.

These findings must be interpreted in light of study limitations. Firstly, the multiple comorbid conditions may influence the significant incidence of causative and potentially causative CNV identified.

Secondly, the small sample size of our cohort, mostly related to the PC-CNV group, combined with the highly skewed sex ratio, with boys greatly outnumbering girls, may introduce several biases in statistical analyses and limit the generalizability of our findings.

Furthermore, despite the array-CGH detecting the presence of CNVs in over half of our cohort, the use of array-CGH alone represents a limitation, as this method cannot detect smaller genetic variants that would be identified by whole-exome or whole-genome sequencing.

Moreover, most of the CNVs identified had uncertain significance. It cannot be excluded that these variants may play a role in susceptibility to the disorder.

Despite these constraints, our study serves primarily as a descriptive and exploratory investigation, providing detailed insights into the genetic architecture of ADHD and relevant indications for clinical practice.

Certainly, larger studies are needed to better clarify the genetic profile and the mechanisms through which genetic risk variants contribute to ADHD pathogenesis, with the aim to identify new possible therapeutic targets for patients suffering from this disorder.

## Figures and Tables

**Table 1:** Demographic and genetic characteristics of ADHD patients. Abbreviations: ADHD, attention deficit/hyperactivity disorder; PC-CNVs, potentially causative copy number variants; CNVs, copy number variants; F, female; M, male; NC-CNVs, non-causative copy number variants; *n*, number; SD, standard deviation.

	Patients (*n*)	Age (Years) (Mean ± SD)	M–F Ratio	Deletions (*n*)	Duplications (*n*)
A ll	152	9 ± 2.5	128:24	-	-
Patients without CNVs (W-CNV)	71	9.4 ± 2.4	63:8	-	-
Patients with CNVs	81	8.7 ± 2.6	65:16	59	64
Patients with PC-CNVs	13	10 ± 2.7	8:5	11	6
Patients with NC-CNVs	68	8.4 ± 2.4	57:11	48	58

**Table 2:** Clinical and molecular characteristics associated with PC-CNVs in patients with ADHD. https://franklin.genoox.com/clinical-db/home (accessed on 12 December 2024).

Proband	CNVs	Type of CNV	Genes Involved	Comorbidities	Dysmorphic Features	Brain MRI Abnormalities
1	arr[GRCh37] 15q13.2q13.3 (31014508_32914140)×1	Pathogenic	12 coding genes, including MTMR10, OTUD7A, CHRNA7	Intellectual disability	Plagiocephaly, high hairline, thick eyebrows, synophrys, long eyelashes, flattened nasal tip, short philtrum, and low-set ears	Arachnoid cyst at the right anterior frontal convexity
2	arr[GRCh37] 16p12.2(21596650_21837555)×1	Pathogenic	3 coding genes, including OTOA, METTL9	Intellectual disability	Narrow forehead, facial asymmetry, hypertelorism, low-set ears	No brain MRI anomalies
3	arr[GRCh37] 16p12.2(21959891_22430592)×1	Likely pathogenic	8 coding genes, including UQCRC2, POLR3E, PDZD9	Intellectual disability	Wide nasal bridge, abnormal ears	Asymmetry of the lateral ventricles, with increased volume of the left one
4	arr[GRCh37] 1p34.3 (35912039_36364474)×1	Likely pathogenic	8 coding genes, including AGO1, AGO4, KIAA0319L	Speech disorder, specific learning disabilities	Rounded face, anterior cowlick, small eyes	No brain MRI anomalies
5	arr[GRCh37] 22q11.21(18641409_21440514)×3	Pathogenic	48 coding genes involving PRODH, HIRA	Tourette syndrome, specific learning disabilities	Broad forehead, thick eyebrows, epicanthal fold, horizontal eyelid rhymes, low-implantation large ears, tragus hypoplasia, flat filter, protruding columella, ogival palate, thin upper lip, V-finger clinodactyly, finger pads	No brain MRI anomalies
6	arr[GRCh37] 9q33.1(119357327_119727205)×1	Pathogenic	TRIM32, ASTN2	Speech disorder	Triangular facies, high forehead, small chin, and hyperlaxity of the small joints	No brain MRI anomalies
7	arr[GRCh37] 16p11.2(29673954_30190568)×3	Pathogenic	27 coding genes involving PRRT2, YPEL3, TAOK2	Speech disorder	No dysmorphism	Asymmetry of the lateral ventricles, with increased volume of the right one
8	arr[GRCh37] 1q21.1q21.2(146564743_147786706)x1	Pathogenic	9 coding genes involving GJA8, GKA5, CHD1L	Specific learning disabilities	Microcephaly, small forehead, arched eyebrows, hint of synophrys, horizontal palpebral fissures, thin upper lip, and microretrognathia	No brain MRI anomalies
9	arr[GRCh37] 22q11.21(20754422_21440514)×1	Pathogenic	14 coding genes involving LZTR1, PI4KA, CLKL	Specific learning disabilities	Abnormal ears and prominent forehead	No brain MRI anomalies
10	arr[GRCh37] 22q11.21(20754422_21440514)×1	Pathogenic	14 coding genes involving LZTR1, PI4KA, CLKL	Specific learning disabilities, Tourette syndrome	Abnormal ears and prominent forehead	No brain MRI anomalies
11	arr[GRCh37] 6q26(161878327_162683777)×1	Pathogenic	PRKN	Specific learning disabilities, Tourette syndrome	No dysmporphism	No brain MRI anomalies
12	arr[GRCh37] 1q21.1(146324068_147786706)×3,16p13.12(13201755_13722409)×1	Pathogenic	10 coding genes involving CHD1L, GJA5, GJA8	Tourette syndrome, obsessive–compulsive disorder	No dysmporphism	Moderate enlargement of the retrocerebellar cerebrospinal fluid space in the posterior median-paramedian left region. Slightly lower position of the cerebellar tonsils
13	arr[GRCh37] Xp22.11p21.3(24156222_25173416)×2,6q26(162799322_162960170)×3,11p15.4(4393574_4832231)×3	Pathogenic	7 coding genes involving ARX, ZFX, PDK3	Specific learning disabilities, tic disorder	Microcephaly, small forehead, big and prominent ears	No brain MRI anomalies

**Table 3:** Somatic and neuropsychiatric features in patients with ADHD according to CNVs. Abbreviations: PC-CNVs, causative copy number variants; CNVs, copy number variants; NC-CNVs, non-causative copy number variants; W-CNVs, without copy number variations; IQ, intelligence quotient; EEG, electroencephalography; MRI, magnetic resonance imaging. Superscript “a” indicates results obtained with the chi-square (χ^2^) test; superscript “b” indicates results obtained with Fisher’s exact test, comparing patients with CNVs and patients without CNVs; superscript “c” indicates results obtained with one-way analysis of variance (ANOVA). Data are shown as means and ± standard deviations or count data and percentages. Statistically significant values (*p*-value < 0.05) are highlighted in bold. The *p*-value of brain MRI anomaly differences remains statistically significant even after the Bonferroni correction.

	PC-CNVs (13)	NC-CNVs (68)	W-CNVs (71)	*p*-Value
D ysmorphic Features	9 (69.2%)	28 (41.2%)	35 (49.3%)	0.162 ^a^
Oppositional Defiant Disorder	1 (7.7%)	14 (20.6%)	20 (28.2%)	0.222 ^a^
Developmental Language Disorder	5 (38.5%)	27 (39.7%)	14 (19.7%)	**0.030** ^a^
Intellectual Disability	3 (23.1%)	15 (22.1%)	13 (18.3%)	0.834 ^a^
Autism Spectrum Disorder	0	16 (23.5%)	9 (12.7%)	0.056 ^a^
Border Intellectual Functioning	2 (15.4%)	6 (8.8%)	9 (12.7%)	0.680 ^a^
Specific Learning Disabilities	7(53.8%)	15 (22.1%)	18 (25.4%)	0.056 ^a^
Tic Disorder	5 (38.5%)	19 (27.9%)	17 (23.9%)	0.54 ^a^
Epilepsy	1 (7.7%)	1 (1.5%)	1 (1.4%)	>0.9999 ^b^
Developmental Coordination Disorder	0	19 (27.9%)	20 (28.2%)	0.086 ^a^
IQ	76.15 ± 24.18	82.32 ± 16.47	83.46 ± 15.68	0.340 ^c^
EEG Anomalies	2 (15.4%)	8 (11.8%)	7 (9.8%)	0.827 ^a^
Brain MRI Anomalies	5 (38.5%)	6 (8.8%)	4 (5.6%)	**0.001** ^a^

**Table 4:** Child Behavior Checklist (CBCL) and Conners’ Parent Rating Scale (Revised): Short Form (CPRS–R:S) subscales T-scores according to CNVs. Abbreviations: PC-CNVs, causative copy number variants; CNVs, copy number variants; NC-CNVs, non-causative copy number variants; W-CNVs, without copy number variations; CBCL Int., Child Behavior Checklist (Internalizing); CBCL Ext., Child Behavior Checklist (Externalizing); CBCL Tot., Child Behavior Checklist Total; CPRS-R Opp., Conners’ Parent Rating Scale (Revised), Oppositional; CPRS-R inatt., Conners’ Parent Rating Scale (Revised). Superscript “d” indicates results obtained with the Kruskal–Wallis test. Data are shown as means and ± standard deviations. Statistically significant values (*p*-value < 0.05) are highlighted in bold and are statistically significant even after the Bonferroni correction.

	PC-CNVs (13)	NC-CNVs (68)	W-CNVs (71)	*p*-Value
C BCL Int. T-score	65.54 ± 14.99	63.87 ± 8.44	66.65 ± 8.9	0.175 ^d^
CBCL Ext. T-score	63.92 ± 10.03	66.4 ± 7.92	69.11 ± 7.3	0.131 ^d^
CBCL Tot. T-score	63.77 ± 9.98	65.9 ± 6.88	67.39 ± 6.47	0.351 ^d^
CPRS-R Opp. T-score	70.31 ± 13.78	68.5 ± 14.07	75.87 ± 15.05	**0.011** ^d^
CPRS-R Inatt. T-score	76.31 ± 15.09	74.4 ± 14.41	83.21 ± 11.07	**0.001** ^d^
CPRS-R Hyperactive T-score	74.38 ± 15.98	75.47 ± 15.15	79.79 ± 15.07	0.149 ^d^
CPRS-R ADHD index	74.62 ± 10.45	75.31 ± 9.58	76.07 ± 13.49	0.581 ^d^

## Data Availability

The data presented in this study are available on request from the corresponding author.

## Abbreviations

The following abbreviations are used in this manuscript:

ADHD	Attention Deficit Hyperactivity Disorder
CNV	Copy Number Variation
MRI	Magnetic Resonance Imaging
EEG	Electroencephalogram
CGH	Comparative Genomic Hybridization
CPRS-R:S	Conners’ Parent Rating Scale (Revised): Short Form
CBCL	Child Behavior Checklist
DSM-5	Diagnostic and Statistical Manual 5
ICD-11	International Classification of Diseases, 11th edition
DLFPC	Dorsolateral Prefrontal Cortex
OCD	Obsessive–Compulsive Disorder
ASD	Autism Spectrum Disorder
SNPs	Single-Nucleotide Polymorphisms
TS	Tourette Syndrome
PC-CNVs	Potential Causative Copy Number Variations
NC-CNVs	Non-causative Copy Number Variations
W-CNVs	Without Copy Number Variations
WISC-IV	Wechsler Intelligence Scale for Children
IQ	Intelligence Quotient
ID	Intellectual Disability
ADI-R	Autism Diagnostic Interview (Revised)
ADOS	Autism Diagnostic Observation Schedule
DDE-2	Dictation and Decoding—2nd Edition
AC-MT	AC—Matematica. Abilità Cognitive in Matematica
MT	MT Reading Tests
DCD	Developmental Coordination Disorder
ABC-2	Autism Behavior Checklist—Second Edition
DGV	Database of Genomic Variants
IRCCS	Istituto di Ricovero e Cura a Carattere Scientifico
OMIM	Online Mendelian Inheritance in Man
VOUS	Variant of Uncertain Significance
SD	Standard Deviation
ANOVA	Analysis of Variance
DLD	Developmental Language Disorder
SLD	Specific Learning Disabilities
ODD	Oppositional Defiant Disorder
BIF	Borderline Intellectual Functioning
